# Longitudinal–Torsional Ultrasonic Grinding of GCr15: Development of Longitudinal–Torsional Ultrasonic System and Prediction of Surface Topography

**DOI:** 10.3390/mi14081626

**Published:** 2023-08-17

**Authors:** Huan Zhang, Ying Niu, Xiaofeng Jia, Shuaizhen Chu, Jingjing Niu

**Affiliations:** 1 School of Mechanical and Power Engineering, Henan Polytechnic University, Jiaozuo 454000, China; 2 School of Mechanical Engineering, Anyang Institute of Technology, Anyang 455000, China

**Keywords:** GCr15, device, surface topography prediction, response surface

## Abstract

The common material of bearing rings is GCr15 bearing steel which is a typical difficult-to-machine material. As an important working surface of the bearing, the inner surface of the raceway plays a vital role in the performance of the bearing. As an important means to solve the high-performance manufacturing of difficult-to-machine materials, longitudinal–torsional ultrasonic processing is widely used in various types of processing. In the presented work, the basic size of the horn is obtained from the wave equation of the forced vibration, and the modal analysis and amplitude test are carried out to verify the rationality of the LUTG structure. Then, according to the probability density function of cutting thickness and the overlapping effect of adjacent abrasive trajectories, the LUTG surface topography prediction model is established by using the height formula of the surface residual material, and the model reliability is verified by using the orthogonal test. The error between the test results and the prediction model is within 13.2%. Finally, based on the response surface method, the optimal process parameters that can meet the requirements of low roughness (Ra) and high material removal rate (MRR) are screened, and the optimal combination of process parameters is obtained as follows: A = 4.5 μm, n = 6493.3 r/min, $a_{p}$ = 28.4 μm, and $v_{f}$ = 21.1 mm/min.

## 1. Introduction

Rolling bearings have the ability to withstand radial loads and single-direction longitudinal loads in rotating mechanisms and are widely used in the aviation and automotive industries and other fields [1,2,3]. The bearing ring is the key component of the rolling bearing. The surface of the bearing ring’s internal circle is an important working surface and plays a vital role in the bearing ring’s performance [4,5]. As a high performance bearing material, Gcr15 bearing steel has high hardness, high strength, and high wear resistance. It is often used in bearing parts such as sleeves and rolling elements. However, GCr15 bearing steel is a typical difficult-to-machine, hard, and brittle material. During processing, if defects such as burns and cracks occur on the surface of the sleeve raceway, they can cause early fatigue and internal surface peeling of the bearing [6,7,8].

Ultrasonic vibration processing technology can reduce the force and heat during processing and improve the surface quality of workpieces. It has shown outstanding advantages in aerospace, the automobile industry, and other technical fields and has become one of the important means to solve the high performance manufacturing of difficult-to-machine materials.

Compared with ordinary processing technology, ultrasonic processing can reduce force and heat during processing and improve the surface quality of the workpiece. Zheng et al. [9] studied ultrasonic vibration grinding of zirconia, established a grinding temperature model, and compared ultrasonic grinding with ordinary grinding tests The results show that ultrasonic vibration can reduce grinding temperature. Xu et al. [10] studied the ultrasonic vibration milling of titanium alloy Tc4 and aluminum alloy 606t6. The test results show that compared with traditional milling, ultrasonic vibration milling can effectively reduce the milling force, reduce the surface defects of the workpiece, and prolong the service life of the tool.

Compared with one-dimensional ultrasonic processing technology, longitudinal–torsional ultrasonic processing technology can further reduce force and heat during processing and improve surface quality [11,12]. Niu et al. [13] used longitudinal–torsional ultrasonic milling to process the difficult-to-machine material titanium alloy and compared the cutting force under ordinary ultrasonic milling and longitudinal–torsional ultrasonic milling via experiments. The results show that the cutting force under longitudinal–torsional ultrasonic milling is lower. Chen et al. [14] studied the longitudinal–torsional and ordinary ultrasonic vibration grinding of silicon carbide ceramics. Under the same grinding conditions, LTUG has a smaller grinding force and lower surface roughness than LUAG.

The surface morphology of the parts has a great influence on the contact state, surface wear, lubrication state, friction, and vibration of the parts. Accurately predicting the surface morphology of the machined surface is of great significance for improving the parts’ quality. Based on the characteristics of ultrasonic vibration processing, Yang et al. [15] proposed a contact velocity model between abrasive particles and workpieces in ultrasonic vibration grinding combined with a single abrasive particle for adjacent abrasive particles. Chen et al. [16] proposed a surface topography modeling and prediction method for ultrasonic grinding considering ploughing. Assuming that the gravel is spherical, a grinding wheel surface model that considered the random distribution of grains was established. Based on the geometric mapping relationship between grains and workpieces in ultrasonic grinding, a grain cutting model considering real-time cutting depth and ploughing action was proposed. Gao et al. [17] designed a new type of ultrasonic vibration grinding device. Assuming that the abrasive grain is a rigid sphere and considering the relationship between the angle of the grinding trajectory and the axis of the workpiece, the radius of the grinding trajectory and the distance between the abrasive grains, a surface topography theoretical model of ultrasonic vibration grinding was established.

The prediction of grinding surface morphology is mostly based on the kinematics and distribution characteristics of abrasive particles, material removal mechanisms, mathematical algorithms, and so on. However, there are few studies on the influence of elastic deformation of the grinding area on surface morphology.

Based on this, this paper proposes a LTUG process for the inner surface of GCr15 bushing. The influence of elastic deformation between the bushing and the grinding wheel on the length of the abrasive cutting path under ultrasonic action is introduced. Based on the longitudinal–torsional ultrasonic vibration, the maximum undeformed chip thickness formula under the action of multiple abrasive grains affected by elastic deformation is established by using the cutting thickness probability density function. The LTUG morphology prediction model is established according to the height value of the surface residual material, and the model is verified. Finally, the surface roughness value is calculated by using the established surface morphology model. The effects of different process parameters and processing conditions on surface roughness and surface morphology are studied.

## 2. Development of LTUG System

**Figure 1:**
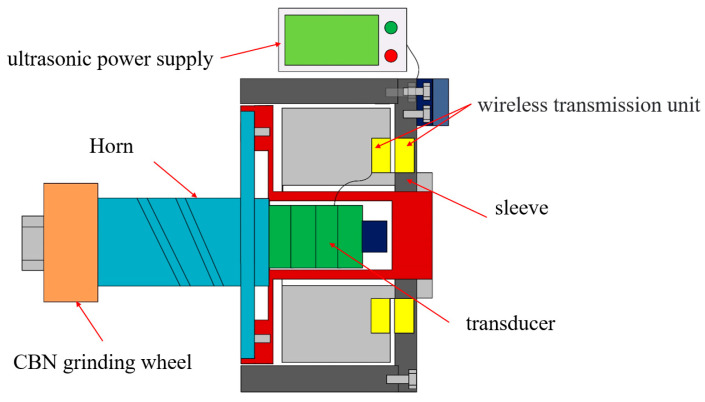
Schematic diagram of the LTUG system.

In the presented work, the grinding wheel is selected as the carrier of vibration. The structure diagram of the ultrasonic vibration grinding system is shown in Figure 1. It is composed of an ultrasonic power supply, transducer, horn, sleeve, wireless transmission unit, and grinding wheel. The ultrasonic power supply converts the alternating current into an ultrasonic frequency electrical signal and transmits the electrical signal to the wireless transmission system. The wireless transmission system transmits the electrical signal to the transducer, and the transducer converts the ultrasonic frequency electrical signal into a sinusoidal mechanical vibration and transmits it to the horn. The horn amplifies the mechanical vibration output by the transducer.

### 2.1. Development of Longitudinal Horn

**Figure 2:**
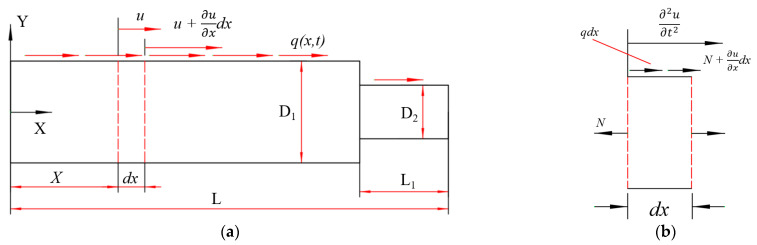
Longitudinal vibration of the horn. (**a**) Longitudinal vibration of an equal straight horn. (**b**) Force analysis diagram of the micro segment.

As shown in Figure 2a, the axial direction of the rod is taken as the *x*-axis, and the infinitesimal segment dx is taken at x on the rod. The axial displacement at the left end is u(x), while the axial displacement at x+dx on the rod is $u+\frac{\partial u}{\partial x}dx$. The deformation in the dx segment is $\frac{\partial u}{\partial x}dx$, while ε represents the strain, and σ represents the stress [18].

The force analysis diagram of the infinitesimal segment dx is shown in Figure 2b. According to Newton’s second law [19]:

(1) $$\rho Adx\frac{\partial^{2}u}{\partial t^{2}}=\left(N+\frac{\partial N}{\partial x}dx\right)-N+q(x,t)dx$$

The above formulas are combined to obtain [20].

(2) $$\frac{\partial^{2}u}{\partial t^{2}}=\left(\frac{E}{\rho }\right)\frac{\partial^{2}u}{\partial x^{2}}+\frac{1}{\rho A}q(x,t)$$

Formula (4) is the wave equation of the forced vibration of the rod. In the formula, $\frac{E}{\rho }=a^{2}$ represents the longitudinal propagation speed of the elastic wave along the rod. The energy exists in the form of a wave during the transmission process. The full wavelength is $\lambda =\frac{a}{f}$ where *f* represents the design frequency. The transmission form of the wave in the rod is a sine curve, and if the horn is too long, it will lead to bending deformation due to insufficient stiffness of the rod; if it is too short, it cannot guarantee that the spindle can completely process the inner circle of the sleeve. Therefore, a wavelength is selected as the boundary condition of the wave equation. Because of the good processing performance and vibration energy transmission effect of 45 steel, 45 steel is selected as the horn material. The design frequency of the horn is 28 kHz.

According to the above formula and analysis, the size of the horn is obtained as shown in Table 1, and the model is imported into the analysis software ANSYS 2021R1. The modal analysis of the model is carried out to verify the rationality of the geometric structure of the horn.

**Figure 3:**
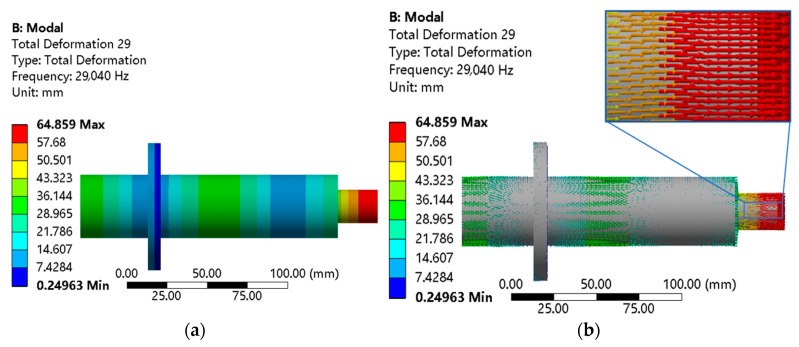
Modal analysis of the longitudinal horn. (**a**) Total deformation cloud image. (**b**) Total deformation vector diagram.

It can be seen from Figure 3 that the vibration effect at the flange is the smallest, and the vibration effect at the small end of the grinding wheel is the largest, showing a better vibration mode. This is because during the transmission of vibration energy in the horn, the small end is small in size, and the energy is concentrated. It can be seen from the above that the horn presents a longitudinal vibration mode, and the deviation between the natural frequency and the design frequency is small, so the structural design of the horn is more reasonable.

### 2.2. Design of Longitudinal–Torsional Ultrasonic Horn

**Figure 4:**
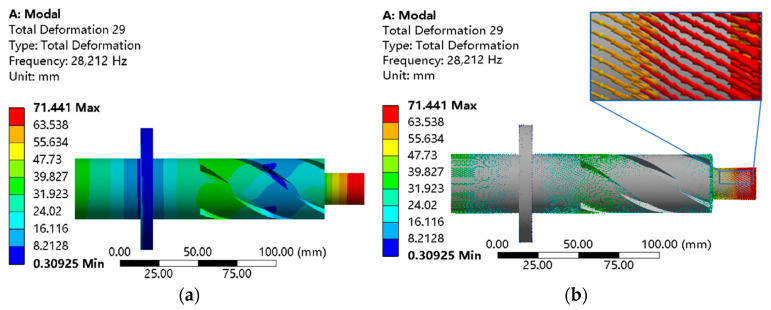
Modal analysis of the longitudinal–torsion horn. (**a**) Total deformation cloud image. (**b**) Total deformation vector diagram.

Adding a spiral groove with a certain geometric structure on the horn can produce a certain amplitude of circumferential vibration. Four spiral grooves with a spiral angle of 45°, a width of 6 mm, and a vertical length of 90 mm are added. The specific simulation results are shown in Figure 4. It can be known from the vibration mode vector that the vibration vector direction of the horn with four spiral grooves corresponds to the change in direction of the spiral groove, resulting in obvious torsional vibration.

**Figure 5:**
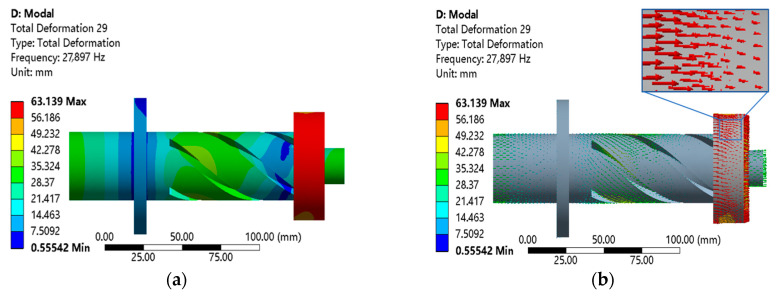
Modal analysis of the LTUG device. (**a**) Total deformation cloud image. (**b**) Total deformation vector diagram.

After obtaining the horn with the determined geometric structure, the models of the longitudinal–torsional ultrasonic horn and the grinding wheel are assembled to obtain the 3D model of the longitudinal–torsional ultrasonic horn. In order to prevent excessive consumption of ultrasonic vibration energy in the working process, the aluminum alloy with a lighter weight is used as the grinding wheel matrix. The model is imported into the analysis software ANSYS, and the modal simulation results are shown in Figure 5.

From Figure 5, it can be seen that the resonant frequency of the ultrasonic amplitude transformer is 27,897 Hz, which is very close to the design frequency. The vibration at the flange of the longitudinal torsional ultrasonic vibration grinding system is the weakest, and the vibration at the front end of the grinding wheel is the strongest, which is in line with the expected effect.

### 2.3. Amplitude Test of LTUG System

**Figure 6:**
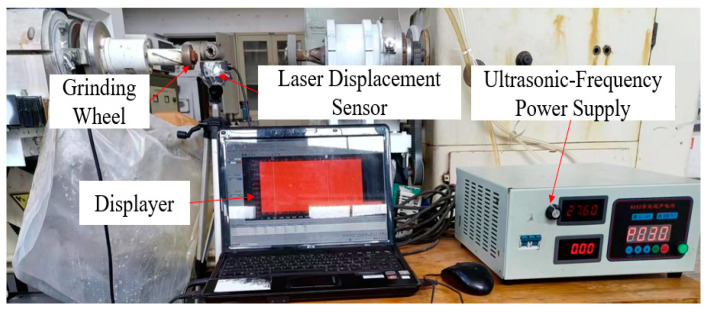
On-site ultrasonic amplitude measurement.

**Figure 7:**
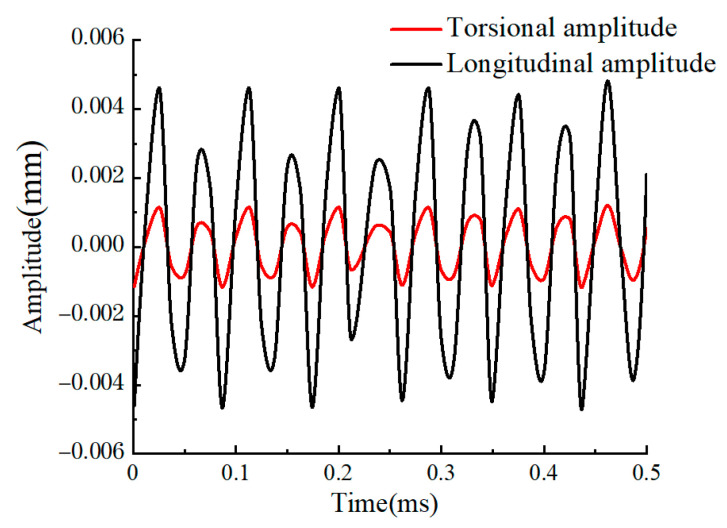
LTUG system amplitude test results.

The amplitude of the LTUG system was measured using a laser displacement sensor. The ultrasonic amplitude measurement site is shown in Figure 6. Four places were evenly selected in the direction of the end face and the circumferential direction of the grinding wheel to measure the longitudinal and torsional vibration amplitudes in both directions. The average value was used as the vibration amplitude of the longitudinal–torsional ultrasonic vibration grinding device. After measurement, it is known that the torsional vibration amplitude at the same frequency is about 25% of the longitudinal amplitude (after averaging the measured data, as shown in Figure 7). After analyzing the amplitude of the LTUG system at the same frequency and with multiple tests of the longitudinal and torsional amplitude of the grinding wheel, it is known that the torsional amplitude of each point in the circumferential direction of the grinding wheel is about 25% of the longitudinal amplitude.

## 3. Surface Topography Prediction of LTUG

At present, the prediction of grinding surface morphology is mostly based on the kinematic trajectory of abrasive particles, material removal mechanisms, the distribution characteristics of abrasive particles, and mathematical algorithms [21], and the influence of elastic deformation between workpieces and grinding wheels on the prediction results is not considered. In this paper, the influence of elastic deformation between the shaft sleeve and the grinding wheel on the length of the cutting path of the abrasive particles under the action of longitudinal torsional ultrasound is introduced. Using the probability density function of the cutting thickness, the formula of the maximum undeformed chip thickness under the action of multiple abrasive particles affected by elastic deformation is established. According to the height of the surface residual material, the LTUG morphology prediction model is established.

In the process of grinding, due to the uneven distribution of abrasive particles on the surface of the grinding wheel and the irregular contour of the abrasive particles, the abrasive particles will process the surface of the workpiece by scraping, ploughing, and cutting. In order to facilitate the modeling, it is assumed that the size and distribution of the abrasive particles on the grinding wheel are uniform and the protrusion height of the abrasive particles is the same, ignoring the effects of the abrasive ploughing and the material fracture on the surface morphology and ignoring the wear of the abrasive particles.

### 3.1. Maximum Undeformed Cutting Thickness Model Considering Elastic Deformation

In the grinding area, the influence of elastic deformation on the arc length of single abrasive grain grinding is mainly divided into two aspects: (1) the additional length $l_{c}^{\prime }$ which is caused by the elastic deformation between the abrasive grain and the workpiece and (2) the additional length $a^{\prime }$ which is caused by the elastic deformation between the grinding wheel and the workpiece. In order to calculate the actual contact arc length affected by the elastic deformation under the action of longitudinal–torsional ultrasonic internal grinding, it is necessary to calculate the additional contact arc length caused by the two factors according to the elastic deformation relationship between the grinding wheel, the abrasive grain, and the workpiece.

The elastic deformation relationship between the abrasive particles and the workpiece is shown in Equation (3), where $K_{i}=\frac{1-u_{i}^{2}}{\pi E_{i}}\;and\;i=g,\zeta$ represent the abrasive particles and the workpiece, respectively. $E_{i}$ and $u_{i}$ represent the elastic modulus and Poisson’s ratio of the two materials, respectively. $F_{n}$ is the normal grinding force of a single abrasive particle under ultrasonic grinding.

(3) $$\delta^{\prime }=\sqrt[{3}]{{\left(\frac{3\pi }{2\sqrt{2}}\right)}^{2}{\left(K_{\zeta }+K_{g}\right)}^{2}(\frac{1}{d_{g_{max}}})F_{n}^{2}}$$

The total additional length caused by elastic deformation is:

(4) $$l_{f}≅l_{c}^{\prime }+a^{\prime }$$

where $l_{c}^{\prime }≅\sqrt{2r\delta },\;a^{\prime }=\sqrt{5.12(\frac{\pi r}{b})(K_{w}+K_{s})F_{n}}$.

Assume:

(5) $$\phi =\sqrt[{3}]{{\left(\frac{3\pi }{2\sqrt{2}}\right)}^{2}{\left(K_{w}+K_{g}\right)}^{3}(\frac{1}{d_{g_{max}}})}$$

(6) $$\psi =\sqrt{5.12(\frac{\pi r}{b})(K_{w}+K_{s})}$$

After finishing, the formula of the arc length of the longitudinal–torsional ultrasonic inner circle contact affected by the elastic deformation is obtained:

(7) $$l=(((\frac{2\sqrt{2\phi r}}{\sqrt[{3}]{bl_{f}C}})F_{n}^{\frac{1}{3}}+\psi F_{n}^{\frac{1}{2}}{)}^{2}+l_{c}{)}^{0.5}$$

The maximum undeformed grinding thickness affected by elastic deformation is:

(8) $$E(h_{1})=\sqrt{\frac{(a_{p}+B)v_{f}}{2Cl{(v}_{s}+v_{g})}}$$

Compared with OG, the trajectories of adjacent abrasive particles in the LTUG grinding area are superimposed. Therefore, the superposition of adjacent abrasive trajectories should be considered when analyzing the maximum undeformed cutting thickness of the LTUG. According to the characteristics of LTUG wear particle motion, the function θ is introduced to define the superposition effect of adjacent wear particle motion trajectories. The expression is shown in Equation (9), where $\xi_{1},\;\xi_{2},\;\xi_{3},\;\xi_{4},\;and\;\xi_{5}$ are the indexes that evaluate the correlation between ϑ and its variables. ϑ is the correlation coefficient between ϑ and its variables.

(9) $$\vartheta =\partial \cdot \frac{v_{s}^{\xi_{4}}\cdot v_{g}^{\xi_{5}}}{{a_{p}}^{\xi_{1}}B^{\xi_{2}}A^{\xi_{3}}}$$

When combining Equations (8) and (9), according to the superposition effect of adjacent abrasive trajectories in the LTUG process, the expected value of the maximum undeformed cutting thickness under the action of multiple abrasive grains is expressed as follows:

(10) $$E(h^{\prime })=\vartheta E(h_{1})=\partial \cdot \frac{v_{s}^{\xi_{4}}\cdot v_{g}^{\xi_{5}}}{f^{\xi_{1}}B^{\xi_{2}}A^{\xi_{3}}}\sqrt{\frac{\left(a_{p}+B)v_{g}\{dg_{max}^{2}{\left(\frac{4\pi }{3\xi }\right)}^{\frac{2}{3}}\right\}}{8wl{(v}_{s}+v_{g})}}$$

The expected value of the maximum undeformed cutting thickness under the action of multiple abrasive grains takes into account many influencing factors, including the influence of the elastic deformation between the abrasive grains, the grinding wheel and the workpiece under the LTUG on the trajectory of the abrasive grains, and the maximum undeformed cutting thickness. In the whole grinding process, there are many abrasive grains involved in grinding. It is necessary to consider the superposition effect of the trajectory of adjacent abrasive grains into the expected value of the maximum undeformed cutting thickness under the action of multiple abrasive grains.

### 3.2. Surface Topography Model of LTUG

**Figure 8:**
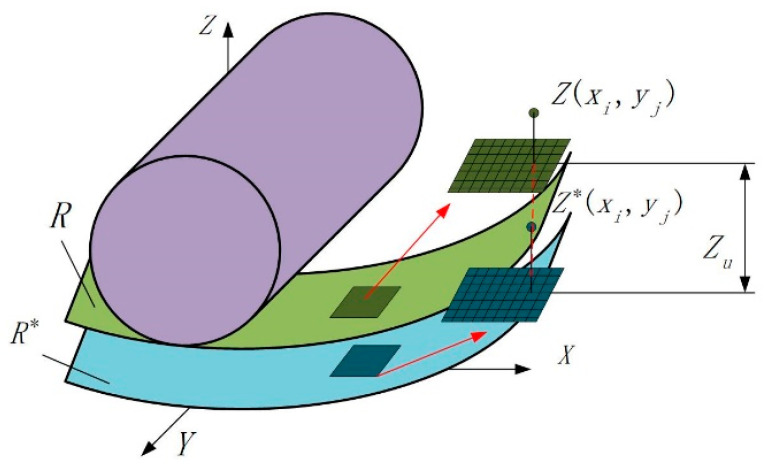
Formation process of the ground surface under the action of random abrasive grains.

In surface prediction model of LTUG, the inner circle is equivalent to a plane at the microscale. The machined surface of high-precision parts is formed under the combined actions of a large number of abrasive particles. These irregularly shaped and different sizes of abrasive particles are irregularly distributed on the surface of the abrasive tool, resulting in the complexity of the material removal mechanism. When the abrasive particles contact the workpiece surface, the abrasive particles act on the grinding area through sliding friction, ploughing, and cutting stages. Figure 8 describes the formation process of the grinding surface under the action of a large number of random abrasive grains from a microscopic point of view. The surface R is the unprocessed surface, and $R^{*}$ is the height of a limited number of points on the unprocessed surface. R decreases when the abrasive grains pass through the grinding contact area, forming the grinding surface $R^{*}$.

**Figure 9:**
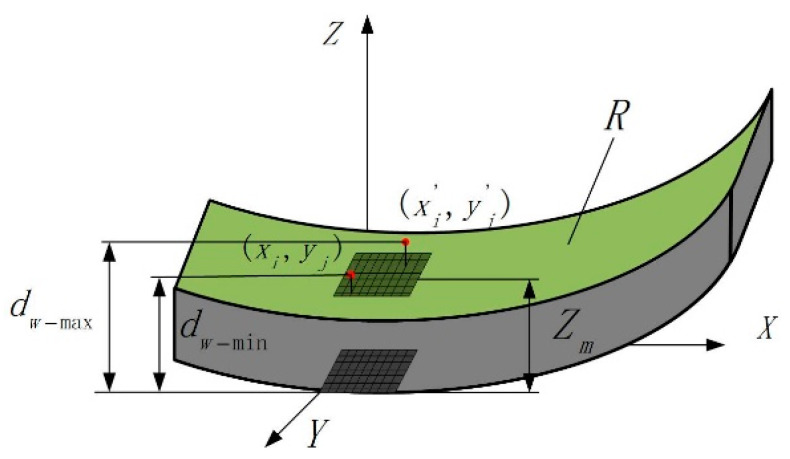
Height and average height of any point on the raw machined surface.

The height of any point on the unprocessed surface of the workpiece and the average height of the unprocessed surface are shown in Figure 9. In order to describe the unprocessed surface before grinding, *Z*_m_ is defined as the average height of the workpiece surface from the XOY surface before grinding, $d_{w-max}$ is defined as the maximum height of the workpiece surface from the XOY surface before grinding, and $d_{w-min}$ is defined as the minimum height of the workpiece surface from the XOY surface before grinding. $Z_{b}\left(x_{i},y_{j}\right)$ can be used to describe the height value of any random point $\left(x_{i},y_{j}\right)$ on the unprocessed surface of the workpiece. According to probability theory, the expression of $Z_{b}\left(x_{i},y_{j}\right)$ is shown in Equation (11), where $\varphi^{\prime }$ is the height deviation of the unprocessed surface of the workpiece.

(11) $$Z_{b}\left(x_{i},y_{j}\right)=Z_{m}+\varphi^{\prime }\;\;\;\varphi^{\prime }\in [-\frac{d_{w-max}-d_{w-min}}{2},\frac{d_{w-max}-d_{w-min}}{2}]$$

According to the superposition of adjacent abrasive grinding trajectories during LTUG, $Z_{u}(x_{i},y_{j})$ is proposed in this study, which means the degree of decline at any point $Z_{b}(x_{i},y_{j})$ on the unprocessed surface of the workpiece under the action of random abrasive $G(x_{i},y_{j})$. The expression is shown in Equation (12), where $l_{g}$ is the length of the grinding contact zone in the direction of the grinding wheel feed speed, $N_{EV}$ is the number of abrasive grains per unit of grinding wheel volume, $a_{p}$ is the grinding depth, and $v_{f}$ is the feed speed.

(12) $$Z_{u}(x_{i},y_{j})=\frac{a_{p}v_{f}}{\vartheta E(h^{\prime })N_{EV}bl_{g}{(v}_{s}+v_{g})}$$

The expression of $N_{EV}$ is shown in Formula (13). $V_{t}$ is based on the percentage of the abrasive volume of the grinding wheel, and $\Gamma =d_{g_{max}}-d_{g_{min}},$ $d_{g_{min}}$ is the minimum diameter of the abrasive. $d_{gx}$ is the diameter of a specific abrasive particle, and the diameter of the abrasive particle obeys the normal distribution.

(13) $$N_{EV}=\frac{3V_{t}\Gamma \sqrt{2\pi }}{4.4\pi \int_{-\Gamma /2}^{\Gamma /2}d_{gx}^{3}exp[-\frac{1}{2}{\left(\frac{4.4}{\delta /2}x\right)}^{2}]dx}$$

The height of the residual material *Z*_a_(*x*_i_, *y*_j_) on the surface of the LTUG is obtained by combining Formulas (11)–(13); that is, the prediction model of the LTUG surface morphology, as shown in Formula (14).

(14) $$Z_{a}\left(x_{i},y_{j}\right)=Z_{b}\left(x_{i},y_{j}\right)-Z_{u}\left(x_{i},y_{j}\right)=Z_{m}-\frac{a_{p}v_{f}}{\vartheta E(h^{\prime })N_{EV}bl_{g}v_{s}}+\varphi^{\prime }$$

## 4. The Surface Morphology Model Test of Gcr15 Bearing Ring Internal Circle in LTUG

### 4.1. Test Conditions and Measurement Methods

**Figure 10:**
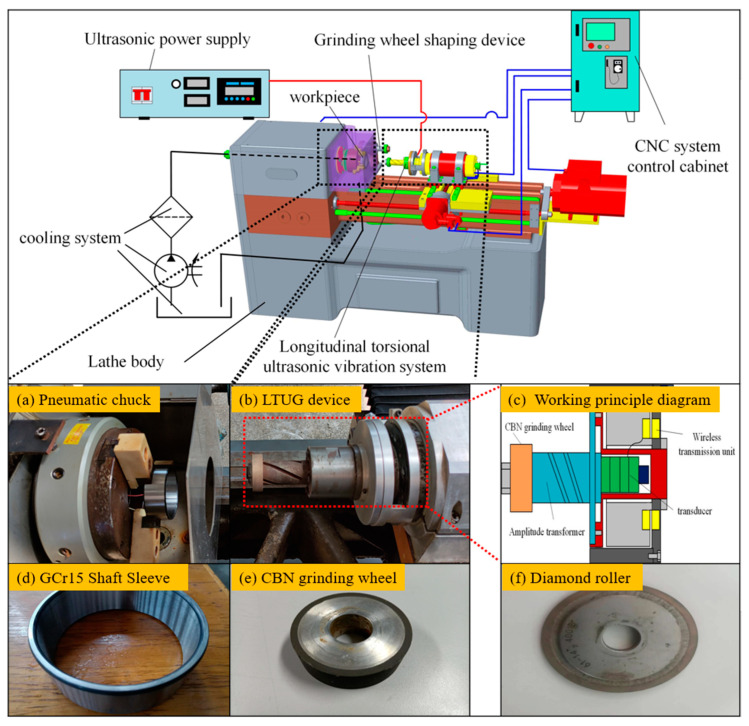
CNC longitudinal torsional ultrasonic internal grinder.

In this test, a CNC internal grinder modified by an ordinary lathe is used. The schematic diagram is shown in Figure 10, which is mainly composed of the CNC system, lathe body, grinding wheel dressing device, and LTUG system. Figure 10a,b shows the LTUG test device. It can be seen that the workpiece is fixed by a special three grab chuck. Figure 10c is the working principle diagram of the ultrasonic structure. The motion of the LTUG system is controlled by the CNC system. When the workpiece is processed, the rotation direction of the motorized spindle is opposite to the rotation direction of the workpiece. The motorized spindle feeds radially towards the workpiece, and the roller can round and sharpen the grinding wheel.

As shown in Figure 10d, the workpiece is a bearing ring with an outer diameter of 110 mm, an inner diameter of 100 mm, and a width of 30 mm. The material is GCr15 bearing steel. Because GCr15 bearing steel has the characteristics of high hardness, high strength, and high wear resistance, the vitrified bond CBN grinding wheel is used in this test. Because of the light weight of aluminum alloy, it is convenient for the vibration energy transfer of the horn. The grinding wheel matrix is selected, and the grain size is 140–170#, as shown in Figure 10e. A metal bond diamond roller is used (as shown in Figure 10f)

After grinding, the workpiece is cut into blocks by an electric spark wire cutting machine. In order to facilitate the observation of the grinding surface morphology and the measurement of its surface roughness, the surface dirt is removed by ultrasonic cleaning with anhydrous ethanol. Then, the surface roughness measuring instrument is used to measure the surface roughness along the grinding’s normal direction. The same position of the grinding surface is measured five times, and the average roughness value is taken as the surface roughness value under the group of processing parameters. The three-dimensional structure and linear contour of the workpiece surface are observed by using a laser confocal scanning microscope.

### 4.2. Test Verification of Surface Topography Prediction Model of LTUG

**Figure 11:**
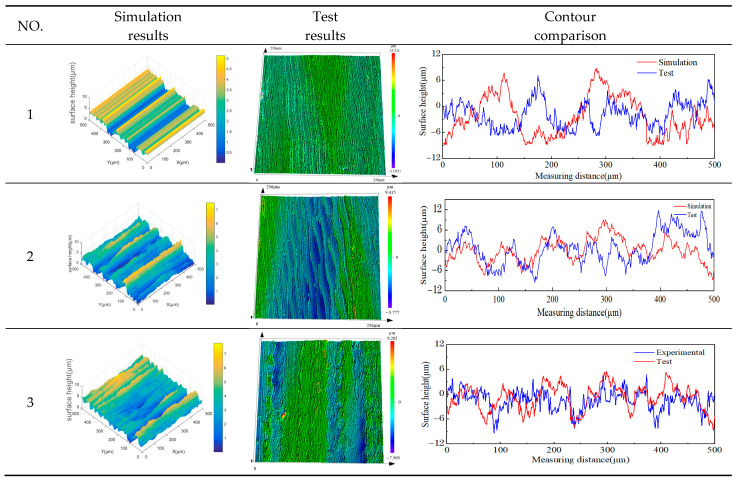

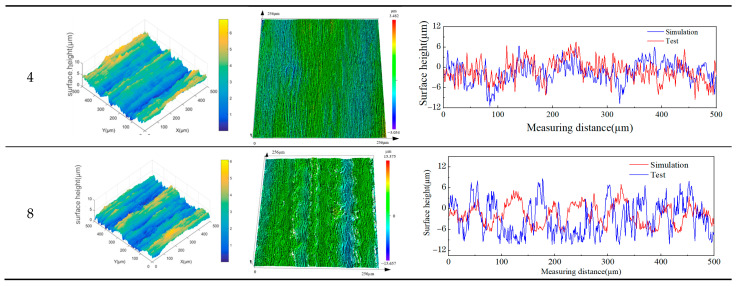
Comparison of simulation and test surface morphology.

In order to verify the accuracy of the surface topography prediction model, the workpiece speed is set to 200 r/min, and the LTUG orthogonal test is performed using the grinding parameters shown in Table 2. The LTUG surface topography prediction model Formula (14) is written into a simulation program using MATLAB 2022a software. The data in Table 2 are brought into the surface topography model, and the partial three-dimensional topography simulation results and test results are obtained as shown in Figure 11.

The output amplitude of the system is changed by adjusting the frequency and output power of the ultrasonic power supply. The output frequency and current of the ultrasonic power supply fluctuate in a small range to ensure the stability of the output amplitude of the system.

Figure 11 shows the three-dimensional morphology simulation and test diagram under the processing parameters in Table 2 and the three-dimensional morphology diagram taken by the confocal microscope and its normal direction contour diagram. It can be seen from the figure that when *A* = 0, the three-dimensional surface morphology of OG has an obvious straight groove microstructure along the cutting direction. With an appropriate increase in the ultrasonic amplitude, the trajectory of the abrasive particles on the machined surface is superimposed, the distribution of the peak and trough on the machined surface becomes more uniform, and the three-dimensional surface morphology of the LTUG has an obvious periodic sinusoidal microstructure. By comparing the three-dimensional morphology of the two processing methods, it can be found that the three-dimensional fluctuation shape of OG is chaotic and irregular, the three-dimensional morphology fluctuation form of the LTUG rises and falls periodically, and the fluctuation range is lower than that of OG, which is related to the sine wave cutting trajectory of the LTUG abrasive particles. The fluctuation law of the simulation graph and the test measurement graph is basically the same. With the change in the ultrasonic amplitude, the spacing between the peaks of the contour also increases. However, when the ultrasonic amplitude *A* = 4 μm, the surface morphology is significantly different from other grinding morphologies. This is due to the higher vibration energy which leads to larger material shaping protrusions and grooves on the machined surface and increases the surface roughness value.

**Figure 12:**
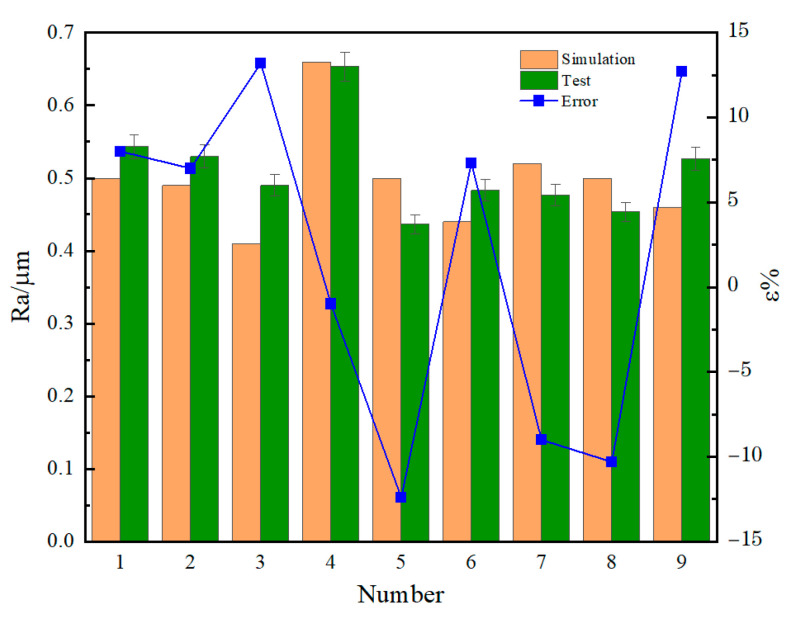
Test and predicted roughness Ra.

The two-dimensional contour is extracted from the three-dimensional topography of Figure 11, and the surface roughness value is obtained by using the root mean square method. For each set of data, the actual roughness value is the average of the roughness values of the five grinding areas. The test results are shown in Figure 12. This prediction model can predict the test results, and the prediction accuracy is within 13.2%.

$$\varepsilon \%\;\mathrm{representation}\;\mathrm{error},\;error=\frac{test-simulation}{test}\times 100\%$$

### 4.3. Multi-Objective Parameter Optimization and Verification Based on Response Surface Method

The advantage of the response surface method is that the various levels of the test factors can be continuously analyzed during the optimization of the test strips. According to the response surface diagram, the influence of each variable on the response factors can be visually observed [22]. Under the constraints of the response factors, relevant software can be used to analyze the test parameters to obtain the optimal parameter combination that satisfies the constraints of the response factors. Therefore, the response surface method is used to design experiments that study the effects of parameters and their interactions on surface roughness and material removal rates [23,24].

The test conditions for the material removal rate are as follows: an electronic balance with high precision is selected as the measuring instrument. Before and after each grinding test, anhydrous ethanol ultrasonic cleaning is used to remove impurities and wear debris on the surface. After the water evaporates, the quality of the workpiece is measured. The quality of each workpiece is measured five times, and the average value is taken as the measurement result of the workpiece. The material removal rate is calculated according to the quality of the workpiece before and after grinding. Using the roughness value and the material removal rate as the corresponding factors, the corresponding relationship between the test parameters and the response factors is shown in Table 3.

Because there are many design variables in this paper and the multivariate quadratic regression equation has 95% accuracy, this paper directly uses the multivariate quadratic regression equation to fit the test data and results of Table 3 and considers all the quadratic terms, primary terms, and interactions between the factors. The curve fitting model of response factor Ra and MRR is obtained using Design-Expert 12 software:

(15) $$\mathrm{Ra}=0.06347+0.08778\;\times \;a_{p}+0.01342\;\times \;v_{f}+1.26333\;\times \;A-5.84331\;\times \;n+16.73851\;\times \;na_{p}+6.66667\;\times \;nA-0.07333\;\times \;nv_{f}+0.00523\;\times \;a_{p}A-0.26871\;a_{p}v_{f}+0.08662\;\times \;v_{f}A-0.00315\;\times \;n^{2}+0.06724\;\times \;a_{p}^{2}-1.68634\;\times \;A^{2}-70.7778\;\times \;v_{f}^{2}$$

(16) $$\mathrm{MRR}=-16.03421+7.38367\;\times \;a_{p}+3.00296\;\times \;v_{f}+1.97321\;\times \;A-0.73966\;\times \;n+1.88326\;\times \;na_{p}+0.08325\;\times \;nA-\;0.05507\;\times \;nv_{f}+0.30681\;\times \;a_{p}A-\;0.110766\;a_{p}v_{f}+0.50526\;\times \;v_{f}A-0.77360\;\times \;n^{2}+0.53086\;\times \;a_{p0}^{2}-0.66773\;\times \;A^{2}-0.35677\;\times \;v_{f}^{2}$$

**Figure 13:**
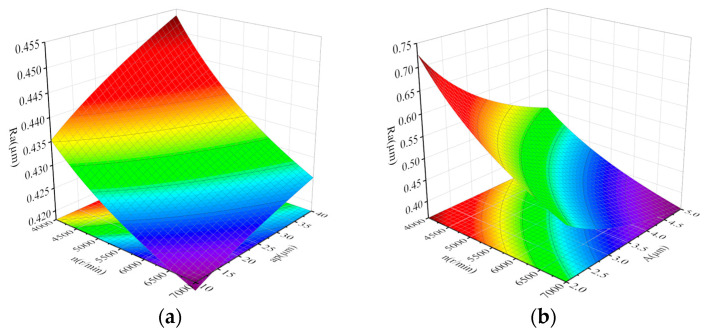

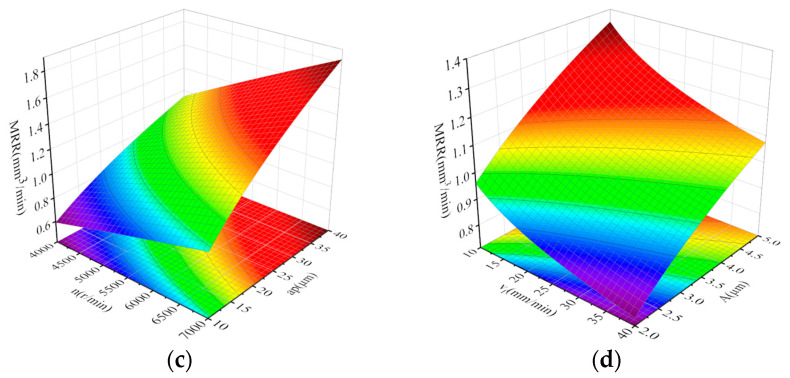
Response surfaces of Ra and MRR corresponding to process parameters. (**a**) Ra for $n,a_{p}$. ($v_{f}$ = 30 mm/min, A = 3 µm). (**b**) Ra for n,A. ($v_{f}$ = 30 mm/min, $a_{p}$ = 40 µm). (**c**) MRR for $n,a_{p}$. ($v_{f}$ = 30 mm/min, A = 3 µm). (**d**) MRR for $v_{f},A$. (n = 5000 r/min, $a_{p}$ = 30 µm).

According to Formulas (15) and (16), the combination of the interaction between design variables that has the greatest influence on the response factor Ra is $na_{p}$, followed by nA. For the interaction between design variables, the combination with the greatest impact on the response factor MRR is $na_{p}$, followed by $v_{f}A$. The response surface of the interaction between the design variables to the response factors is shown in Figure 13.

Figure 13a is the response surface obtained under the condition of $v_{f}$ = 30 mm/min and *A* = 3 μm. From the diagram, it can be seen that Ra increases with the increase in the grinding wheel speed and decreases with the increase in the grinding depth. Compared with the grinding depth, the influence of the grinding wheel speed on Ra is greater. Figure 13b is the response surface obtained under the condition of $v_{f}$ = 30 mm/min and $a_{p}$ = 40 μm. From the graph, it can be seen that Ra increases with the increase in the grinding wheel speed, and with the increase in the amplitude, the downward trend of Ra slows down. Compared with the amplitude, the influence of the grinding wheel speed on Ra is greater. Figure 13c is the response surface obtained under the condition of $v_{f}$ = 30 mm/min and *A* = 3 μm. It can be seen from the figure that MRR increases with the increase in the grinding wheel speed and grinding depth. Compared with the grinding wheel speed, the grinding depth has a greater influence on Ra. Figure 13d is the response surface obtained under the condition of n = 5000 r/min and $a_{p}$ = 30 μm. From the graph, it is known that MRR increases with the increase in amplitude A and decreases with the increase in the feed speed. From the contour line, the influence of the two parameters on MRR is small.

Aiming at low Ra and high MRR, the optimal combination of process parameters obtained by Design-Expert is:

A = 4.5 μm, n = 6493.3 r/min, $a_{p}$ = 28.4 μm, $v_{f}$ = 21.1 mm/min, predicted Ra = 0.472 μm, and MRR = 1.676 mm^3^/min.

**Figure 14:**
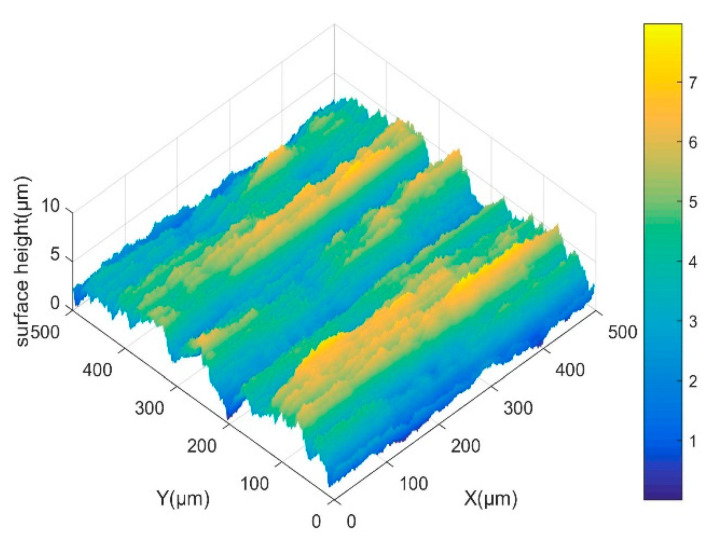
Optimized surface topography simulation.

**Figure 15:**
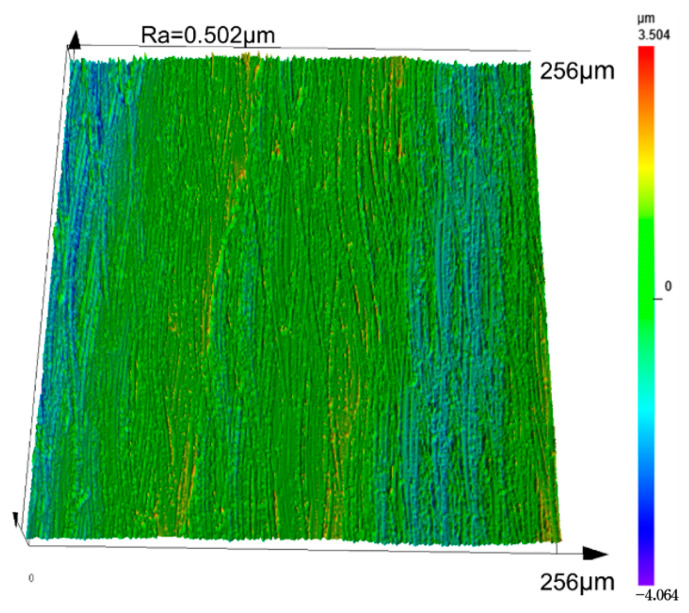
Surface morphology and roughness of the optimized samples.

According to the optimal process parameters obtained by Design-Expert, the surface topography prediction model is introduced to simulate the surface topography, as shown in Figure 14. From the simulation diagram, it can be seen that the peak and trough are evenly distributed, and the peak and trough difference is small. The surface morphology and roughness of the longitudinal–torsional ultrasonic internal grinding test using the optimized parameters are shown in Figure 15. From the figure, it can be seen that the grinding surface morphology has good uniformity and is in good agreement with the simulated morphology. The measured surface roughness of the sample is Ra = 0.502 μm. According to the calculation, the relative error between the actual value of Ra and the predicted value is 5.9%. Because the roughness test and the material removal rate test use the same set of parameters, there is no need to carry out the second test. After calculation, the actual material removal rate is MRR = 1.763 mm^3^/min, and the relative error between the actual value of MRR and the predicted value is 5.1%. The relative error between the actual value and the predicted value of the response factor shows that the optimization method is reliable and has guiding significance for the longitudinal–torsional ultrasonic internal grinding process of GCr15 bearing steel.

## 5. Conclusions

In the presented work, a longitudinal–torsional ultrasonic grinding system was developed, the surface morphology prediction model of LTUG was established, and the process parameters were optimized. The main conclusions are as follows:

(1) A LTUG system was developed. The LUTG system ultrasonic frequency was 28.1 kHz, the longitudinal amplitude was between 0–5 μm, and the torsional amplitude had a longitudinal amplitude of 25%.

(2) Considering the established elastic–plastic deformation longitudinal–torsional ultrasonic grinding surface morphology prediction model of LUTG, the prediction accuracy was within 13.2% when compared with the test.

(3) Aiming at low Ra and high MRR Ra and MMR as the targets, the optimal process parameters were: A = 4.5 μm, n = 6493.3 r/min, $a_{p}$ = 28.4 μm, and $v_{f}$ = 21.1 mm/min.

## Figures and Tables

**Table 1:** Structural parameters of the longitudinal amplitude horn.

Type	L/mm	L1/mm	D1/mm	D2/mm
Parameter	182	33	38	20

**Table 2:** Grinding parameters of the orthogonal test.

Number	Revolution Speed *n* (r/min)	Grinding Depth *a_p_* (um)	Feed Speed *v_f_* (mm/min)	Amplitude *A* (µm)
1	4000	10	10	0
2	4000	30	30	2
3	4000	50	50	4
4	6000	10	30	2
5	6000	30	50	4
6	6000	50	10	0
7	8000	10	50	4
8	8000	30	10	2
9	8000	50	30	0

**Table 3:** Test plan and response results.

No	$a_{p}$	*n* (r/min)	$v_{f}$ (mm/min)	*A* (µm)	Ra (µm)	MRR (mm^3^/min)
1	10	4000	10	2	0.43	0.61
2	10	5000	20	3	0.68	0.73
3	10	6000	30	4	0.55	0.84
4	10	7000	40	5	0.51	1.02
5	20	4000	20	4	0.48	0.76
6	20	5000	10	5	0.76	0.93
7	20	6000	40	2	0.64	1.15
8	20	7000	30	3	0.39	1.32
9	30	4000	30	5	0.6	0.97
10	30	5000	40	4	0.43	1.28
11	30	6000	10	3	0.78	1.44
12	30	7000	20	2	0.45	1.68
13	40	4000	40	3	0.5	1.47
14	40	5000	30	2	0.65	1.72
15	40	6000	20	5	0.46	1.82
16	40	7000	10	4	0.4	1.76

## Data Availability

The data are unavailable due to privacy.
